# Effect of Carotene and Lycopene on the Risk of Prostate Cancer: A Systematic Review and Dose-Response Meta-Analysis of Observational Studies

**DOI:** 10.1371/journal.pone.0137427

**Published:** 2015-09-15

**Authors:** Yulan Wang, Ran Cui, Yuanyuan Xiao, Juemin Fang, Qing Xu

**Affiliations:** 1 Department of Clinical Laboratory, Shanghai Tenth People's Hospital, Tongji University, School of Medicine, No.301 Middle Yanchang Road, Zhabei District, Shanghai 200072, China; 2 Department of Oncology, Shanghai Tenth People's Hospital, Tongji University, School of Medicine, No.301 Middle Yanchang Road, Zhabei District, Shanghai 200072, China; Van Andel Institute, UNITED STATES

## Abstract

**Background:**

Many epidemiologic studies have investigated the association between carotenoids intake and risk of Prostate cancer (PCa). However, results have been inconclusive.

**Methods:**

We conducted a systematic review and dose-response meta-analysis of dietary intake or blood concentrations of carotenoids in relation to PCa risk. We summarized the data from 34 eligible studies (10 cohort, 11 nested case-control and 13 case-control studies) and estimated summary Risk Ratios (RRs) and 95% confidence intervals (CIs) using random-effects models.

**Results:**

Neither dietary β-carotene intake nor its blood levels was associated with reduced PCa risk. Dietary α-carotene intake and lycopene consumption (both dietary intake and its blood levels) were all associated with reduced risk of PCa (RR for dietary α-carotene intake: 0.87, 95%CI: 0.76–0.99; RR for dietary lycopene intake: 0.86, 95%CI: 0.75–0.98; RR for blood lycopene levels: 0.81, 95%CI: 0.69–0.96). However, neither blood α-carotene levels nor blood lycopene levels could reduce the risk of advanced PCa. Dose-response analysis indicated that risk of PCa was reduced by 2% per 0.2mg/day (95%CI: 0.96–0.99) increment of dietary α-carotene intake or 3% per 1mg/day (95%CI: 0.94–0.99) increment of dietary lycopene intake.

**Conclusions:**

α-carotene and lycopene, but not β-carotene, were inversely associated with the risk of PCa. However, both α-carotene and lycopene could not lower the risk of advanced PCa.

## Introduction

PCa is the second most abundant male cancer [1]. Owing to the improved screening and early detection procedures, rising incidence rates of PCa have been observed over the last few decades [2]. However, the success in treating advanced PCa remains poor, drawing attention to dietary factors that may influence risk of this malignancy, particularly carotenoids [3]. Lots of epidemiological factors, including age, gender, ethnicity, genetic factors, family history, lifestyle, region and diet, have been considered to be associated with the development of PCa [4]. For example, Asian populations are generally at lower PCa risk compared with the Western populations [5]. Moreover, the average annual incidence rate of PCa between 1988 and 1992 among Chinese men in the United States was 15 times higher than that of their counterparts living in Shanghai and Tianjin [6], specially, the change of diet and lifestyle inevitably resulted in the increased prevalence of obesity in East Asia [7], which might be responsible for increasing trend of PCa in East Asia—all of which suggest that variations in lifestyle and diet may play a crucial role in PCa. Among a large number of components of foods, carotenoids, especially its main active ingredients—carotene and lycopene, have received special attention due to its promising antioxidative properties [8–10].

Carotenoids, which include α-carotene, β-carotene, lycopene, β-cryptoxanthin, lutein, and zeaxanthin that represent the major carotenoids in the human diet [8], provide the yellow, orange and red pigments in fruits and vegetables [11]. Carotenoids have distinct antioxidative properties, including protecting DNA and other important biomolecules from free radicals [12]. In 1981, Peto et al. hypothesized that dietary β-carotene from fruit and vegetables may reduce human cancer incidence rates [13], since then a flurry of epidemiologic studies had addressed this topic [14–17]. Carotenes(including α-carotene and β-carotene) have been investigated for many years now, but whether carotenes are related to the PCa are still mostly inconsistent. Lycopene is one of the most effective oxygen radical quenching agents among the carotenoids [18], which is found in relatively high concentrations in the prostate gland [19]. The results of epidemiological studies have generally supported a protective effect on cancer of carotenoid-rich foods. According to the latest Continuous Update Project(CUP) report summarized by the World Cancer Research Fund(WCRF) in November 2014, there is limited evidence for the effect of lycopene on PCa risk [20]. In contrast, substantial intake of β-carotene is unlikely to have any effect on the PCa risk. These inconsistencies could be mainly due to confounding by nutritional as well as non-nutritional factors and lack of validity of carotenoids estimates due to inaccurate dietary or blood concentration assessment.

Based on these inconsistencies, we conducted this meta-analysis on all published epidemiologic studies to date to reevaluate and quantify the relation between dietary intake or blood concentrations of α-carotene, β-carotene, lycopene and risk of PCa.

## Materials and Methods

### Literature search

This meta-analysis was conducted in accordance with PRISMA guidelines(S1 PRISMA Checklist). We conducted a comprehensive literature search of PubMed (http://www.ncbi.nlm.nih.gov/pubmed) and Embase (http://www.elsevier.com/online-tools/embase) (up to January 2015) using the key words: carotenoids, carotene, lycopene, prostate cancer, case-control study, cohort study and text terms: micronutrients. Bibliographies from retrieved articles were also scoured to find further eligible studies.

### Study selection

Our objective was to thoroughly evaluate the relation between dietary intake or blood concentrations of α-carotene, β-carotene, lycopene and risk of PCa. Studies that met the following criteria were included in the meta-analysis: *1*) used epidemiologic investigation design: case-control study, nested case-control study, cohort study, etc; *2*) evaluated the association between carotene(α- and/or β-), lycopene and PCa risk; and *3*) provided RRs with 95% CIs for ≥3 exposure categories. Furthermore, studies that additionally provided the doses of carotenoids, the number of cases, and the number of controls(or person-years) in each of exposure categories were included in the dose-response meta-analysis.

### Data extraction and statistical analysis

The following information was extracted from each study: name of the first author, year of publication, location of study, study period, study type, age of the study population at baseline, number of cases/controls/total participants, year of follow-up, range of exposure(dietary intake or blood levels) and adjustment for covariates. Independent data extraction was performed by two authors(YL W and RC). Any discrepancies were resolved through discussing with the third reviewer(QX).

Given the fact that the quality of the included studies evaluating these relations, especially in terms of statistical power and the rigor with which the dietary data were collected, varied considerably, we conducted a quality assessment on preliminarily included studies, by using the 9-star Newcastle-Ottawa Scale (NOS) [21], which is a validated scale for non-randomized studies in a meta-analysis. This scale includes three aspects of evaluation: the selection of the cohorts, comparability of cohorts, and ascertainment of the exposure and outcome of interest. We regarded scores of 1–3, 4–6, and 7–9 as low, moderate, and high quality, respectively.

A random-effect model was utilized to consider both within-study and between-study variations in RR estimates [22]. As different studies might report different exposure categories, such as dichotomous, thirds, quarters, or fifths, we used the study specific RR for the highest versus lowest category of dietary carotenoids intake(mg/day) or carotenoids concentration(ug/dl) exposure for the meta-analysis. Cochran Q test and I^2^ statistic were used to assess the heterogeneity [23]. We also performed sensitivity analyses to evaluate whether the pooled results could have been markedly affected by sequentially exclude a single study at a time. Subgroup analyses were performed for study type, regions, covariate adjustment.

For the meta-analysis of the dose-response relationship between carotenoids and PCa risk, the method of generalized least squares for trend estimation proposed by Greenland and Longnecker and Orsini et al [24, 25], was performed using restricted cubic splines with 3 knots at percentiles 33%, 66%, and 99% of the distribution. A *P* value for curvelinearity or nonlinearity was calculated by testing the null hypothesis that the coefficient of the second spline was equal to zero. We used the Stata 12(Stata Corp., College Station, Texas) to perform all statistical tests. *p*<0.05 was considered statistically significant.

## Results

### Literature search

**Fig 1:**
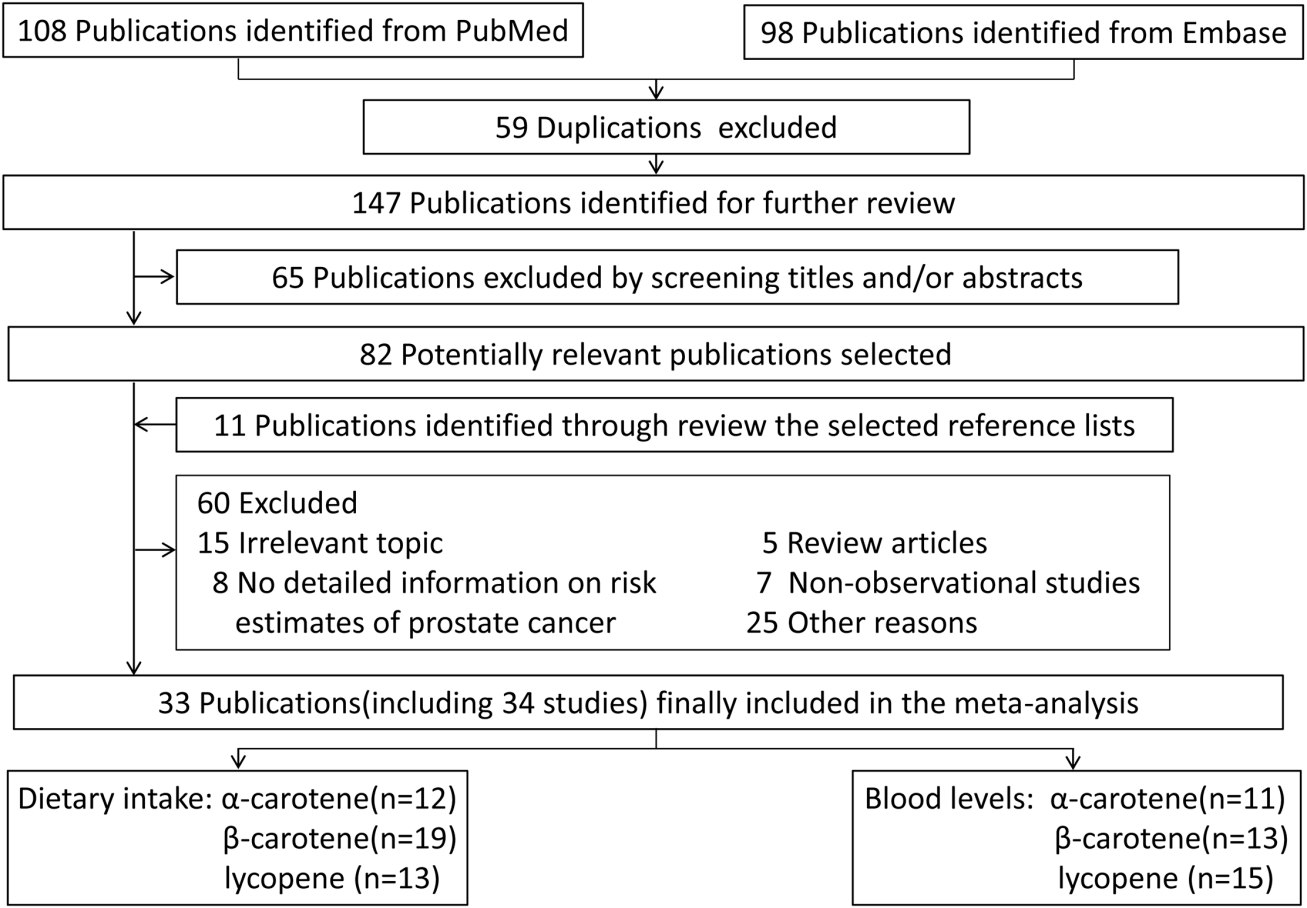
The literature search process.

The initial screening yielded 206 publications. After selection, a total of 34 studies (1 article [26] reported results from two subcohorts) from 33 publications [14–17, 26–54] were included in the meta-analysis. Among these studies, twelve, nineteen and thirteen studies reported the effects of dietary intake of α-carotene, β-carotene, lycopene on PCa risk, respectively. Eleven, thirteen and fifteen studies reported the effects of blood levels of α-carotene, β-carotene, lycopene on PCa risk, respectively(Fig 1).

### Study characteristics

Among these 34 studies, 10 studies were cohort studies (two studies were case-cohort studies), 11 studies were nested case-control studies, and 13 studies were case-control studies(Table 1).

**Table 1:** Characteristics of included studies.

Source, y	Location	Study Period	Study Type	Age, y(SD)	No.of Cases	No.of Matched Controls	No.of Participants	Follow-up,y	Range of Exposure:blood(ug/dl);intake(mg/day)	Adjustment for Covariates
Karppi,2009	Finland	1993–2006	Cohort	56.2	55	—	997	12.6	Serum lycopene:4.68(T1),11.11(T3)	Age, examination year, alcohol intake, FHPC, physical activity, waist-to-hip ratio, education, smoking, and serum folate.
Shibata,1992	USA	1981–1989	Cohort	65–85	1,335	—	11,580	>8	Intake α-carotene:1.8(T1),8.6(T3)	Age and smoking.
Giovannucci,1995	USA	1986–1992	Cohort	40–75	812	—	47,894	6	Intake α-carotene:0.243(Q1),0.221(Q4);β-carotene:2.1(Q1),10.8(Q4);lycopene:1.5(Q1),10.1(Q4)	Age and energy.
Ambrosini,2008	Australia	1990–2004	Cohort	Cases:59.2–66.2^b^ Controls:47.5–62.5^b^	97	—	2,183	12	Intake β-carotene:1.8(T1),4.6(Q4)	Age, fruit and vegetable intake, retinol/β-carotene supplement and crocidolite exposure.
Kirsh,2006	USA	1993–2001	Cohort	55–74	1,338	—	29,361	8	Intake α-carotene:0.47(Q1),2.32(Q5);lycopene:5.05(Q1),15.6(Q5)	Age, energy, race, study center, FHPC,BMI, smoking, physical activity, fat/red meat intake,history of diabetes, and aspirin use.
Umesawa,2013	Japan	1988–1990	Cohort	40–79	143	—	15,471	20	Intake α-carotene:0.11(Q1),0.50(Q5);β-carotene:0.1(Q1),3.72(Q5)	Saturated fat/isoflavone/α-tocopherol intake.
Daviglus,1996	USA	1959–1989	Cohort	40–55	132	—	1,899	30	Intake β-carotene:2.3(Q1),4.0(Q4)	Age, smoking, cholesteroal/saturated fat/ethanol intake, energy, and occupation.
Roswall,2013	Denmark	1993–1997	Cohort	50–64	1,571	—	26,856	7	Intake β-carotene:1.6(Q1),4.71(Q4)	Height, weight, education, red meat/alcohol/selenium intake.
Schuurman,2002	Netherlands	1986–1992	Case-Cohort	55–70	642	1,525	58,279	6.3	NR(intake)	Age, FHPC, socioeconomic status, and alcohol intake.
Agalliu,2001	Canada	2003–2010	Case-Cohort	Cases:66.2(8.4) Subcohort:69.3(10.5)	661	1,864	34,291	7	Intake β-carotene:2.26(Q1),10.77(Q5);lycopene:2.45(Q1),15.87(Q5)	Age, race, BMI, physical activity, and education.
Key,2007	European Countries	1992–2000	NCCS	Cases:60.4(5.8) Controls:60.1(5.8)	966	1,064	137,001	4	Plasma α-carotene:2.59(Q1),10.51(Q5);β-carotene:8.21(Q1),27.28(Q5);lycopene:15.04(Q1),49.37(Q5)	Age, fasting hours prior to blood draw, BMI,FHPC, and education.
Huang,2002	USA	CLUE^a^ I:1974–1996;CLUE II:1989–1996	NCCS	CLUE I:Cases:54 (9) Controls:54 (9) CLUE II: Cases:66 (8) Controls:66 (9)	CLUE I:182;CLUE II:142	CLUE I 364 CLUE II 284	CLUE I:9,804 CLUE II:10,456	CLUE I:17 CLUE II:3.5	CLUE I:Serum α-carotene:1.4(Q1),3.9(Q5);β-carotene:4.4(Q1),15.6(Q5);lycopene:21.7(Q1),54.9(Q5) CLUE II:Serum α-carotene:1.2(Q1),5.5(Q5);β-carotene:4.2(Q1),15.8(Q5);lycopene:24.3(Q1),62.8(Q5)	Age, number of years since blood was drawn, disease stage at diagnosis, smoking, and BMI.
Goodman,2003	USA	1983–1997	NCCS	45–75	205	205	18,314	>10	Serum α-carotene:1.8(Q1),4.3(Q4);β-carotene:9.4(Q1),21.9(Q4);lycopene:22.9(Q1),41.7(Q4)	Age, study center at randomization, sex, smoking, and year of randomization.
Gann,1999	USA	1982–1995	NCCS	40–85	578	1,294	22,071	13	Plasma α-carotene:3.46(Q1),10.33(Q4);lycopene:26.17(Q1),58.01(Q4)	Physical activity, BMI, plasma total cholesterol, alcohol intake, and multivitamin supplement use.
Nomura,1997	USA	1971–1975	NCCS	52–75	142	142	6,860	>20	NR(intake)	NR
Beilby,2010	Australia	Since 1990	NCCS	Cases:69.8(7.2) Controls:69.3(6.7)	96	226	4,890	>10	Serum β-carotene:0.11(T1),1.16(T3);lycopene:0.05(T1),0.43(T3)	Age, and vitamin A supplement.
Peters,2007	USA	1993–2001	NCCS	55–75	692	844	28,243	8	Serum α-carotene:2.6(Q1),16.6(Q5);β-carotene:6.1(Q1),38.7(Q5);lycopene:30.5(Q1),108.4(Q5)	Age, time since initial screening, year of blood draw, and study center.
Wu,2004	USA	1993–1998	NCCS	40–75	450	450	18,259	>5	NR(blood)	Cholesterol levels, selenium/Vitamin E supplementation, FHPC, BMI, height, physical activity, history of vasectomy and current smoking.
Gill,2009	USA	1993–1996	NCCS	45–75	467	936	96,382	>3	Serum β-carotene:9.8(Q1),59.7(Q4);lycopene:22.0(Q1),65.6(Q4)	Age, fasting hours prior to blood draw, BMI, FHPC, and education.
Hsing,1990	USA	1974–1986	NCCS	47–91	103	103	206	13	NR(blood)	Age, race, smoking, education, time of last meal.
Andersson,1996	Sweden	1989–1994	CC	<81	526	536	1,062	—	NR(intake)	Age and energy.
Norrish,1998	New Zealand	1996–1997	CC	40–81	317	480	797	—	Intake β-carotene:2.5(Q1),6.1(Q4);lycopene:0.7(Q1),2.0(Q4)	Age, height, NSAIDs, and socioeconomlc status.
Bosetti,2004	Italy	1991–2002	CC	46–75	1,294	1,451	2,745	—	NR(intake)	Age, study center, education, physical activity, BMI,FHPC and energy.
Mettlin,1989	USA	Since 1987	CC	55–86	371	371	742	—	NR(intake)	Age
Zhang,2007	USA	1998–2003	CC	Cases:64.4(9.0) Controls:59.4(10.5)	193	197	390	—	Plasma α-carotene:0.71(Q1),6.08(Q4);β-carotene:3.47(Q1),28.35(Q4);lycopene:14.05(Q1),51.37(Q4)	Age, race, BMI, education, and smoking.
Jian,2005	China	2001–2002	CC	Cases:72.7(7.1) Controls:71.4(7.2)	130	274	404	—	Intake α-carotene:0.24(Q1),1.79(Q4);β-carotene:1.96(Q1),7.49(Q4);lycopene:1.61(Q1),4.92(Q4)	Age, locality, education, family income, marital status, number of children, FHPC, BMI, tea drinking, energy, fat intake.
Chang,2005	USA	1996–1998	CC	Cases:63.9(7.0) Controls:62.8(6.6)	118	52	170	—	NR(blood)	Age, smoking, and height.
McCann,2009	USA	1986–1991	CC	NA	433	538	971	—	Intake α-carotene:0.63(Q1),1.5(Q4);β-carotene:3.8(Q1),8.04(Q4);lycopene:3.9(Q1),8.86(Q4)	Age, education, BMI, smoking, and energy.
Meyer,1997	Canada	1990–1993	CC	>45	215	593	808	—	NR(intake)	Age, education, FHPC, energy.
Jain, 1999	Canada	1989–1993	CC	Cases:69.8 Controls:69.9	617	636	1,253	—	Intake α-carotene:0.84(Q1),2.16(Q4);β-carotene:3.0(Q1),7.83(Q4);lycopene:2.1(Q1),12.67(Q4)	Age, energy, vasectomy, smoking, marital status, study area, BMI, vitamin use, diet.
Deneo-Pelligrini,1999	Uruguay	1994–1997	CC	40–89	175	240	415	—	Intake α-carotene:0.11(Q1),0.60(Q4);β-carotene:2.71(Q1),7.49(Q4);lycopene:1.3(Q1),3.3(Q4)	Age, residence, education, FHPC, BMI, energy.
Cohen,2000	USA	1993–1996	CC	40–64	628	602	1,230	—	Intake α-carotene:0.33(Q1),0.81(Q4);β-carotene:2.2(Q1),4.4(Q4);lycopene:4.9(Q1),9.9(Q4)	Age, race, fat intake, energy, FHPC, BMI, antigen tests, education.
Lu,2001	USA	1993–1997	CC	Cases:59.98(6.19) Controls:41.9(13.64)	65	130	195	—	Intake α-carotene:0.39(Q1),1.14(Q4);β-carotene:2.38(Q1),5.85(Q4);lycopene:1.46(Q1),3.45(Q4) Plasma α-carotene:2.02(Q1),5.85(Q4);β-carotene:7.25(Q1),19.9(Q4);lycopene:10.45(Q1),23.39(Q4)	Age, race, smoking, education, FHPC, alcohol intake, energy.

Abbreviations: NCCS, nested case-control study; CCS, case-control study; SD, standard deviation; T, tertile; Q, quartile/quintile; BMI, body mass index; NSAIDs, non-steroidal anti-inflammatory drugs; FHPC, family history of prostate cancer; NR, not reported; NA, not accessible.

^a^Derived from the slogan of a campaign, “Give us a CLUE to cancer.”

^b^Indicated interquartile range(IQR).

They involved a total of 15,891 cases and 592,479 participants. Twenty two studies were from the North America, 7 studies were from the Europe, 2 studies were from Australia, 2 study were from the Asian countries, and 1 study was from Uruguay. With respect to the dietary intake of carotenoids, 12 studies studied on α-carotene, 19 studies studied on β-carotene, and 13 studies studied on lycopene. With respect to the blood levels of carotenoids, 11 studies studied on α-carotene, 13 studies studied on β-carotene, 15 studies studied on lycopene. All these included observational studies utilized structured food frequency questionnaire to collect participants’ information on usual food consumption.

Most studies provided risk estimates that were adjusted for age (29 studies); few adjusted for smoking(14 studies), body mass index(BMI)(15 studies), family history of PCa(FHPC)(12 studies), energy intake(12 studies), alcohol intake(3 studies), physical activity (6 studies) and education(14 studies). All studies but nine studies [15, 16, 29, 35, 42, 43, 45, 49, 53] provided ranges of exposure in each of exposure categories. The mean NOS score was 7.6 stars (range, 4–9 stars; S1 Table), suggesting that the study quality was fair.

### Dietary intake of α-carotene, β-carotene, lycopene and PCa risk

**Fig 2:**
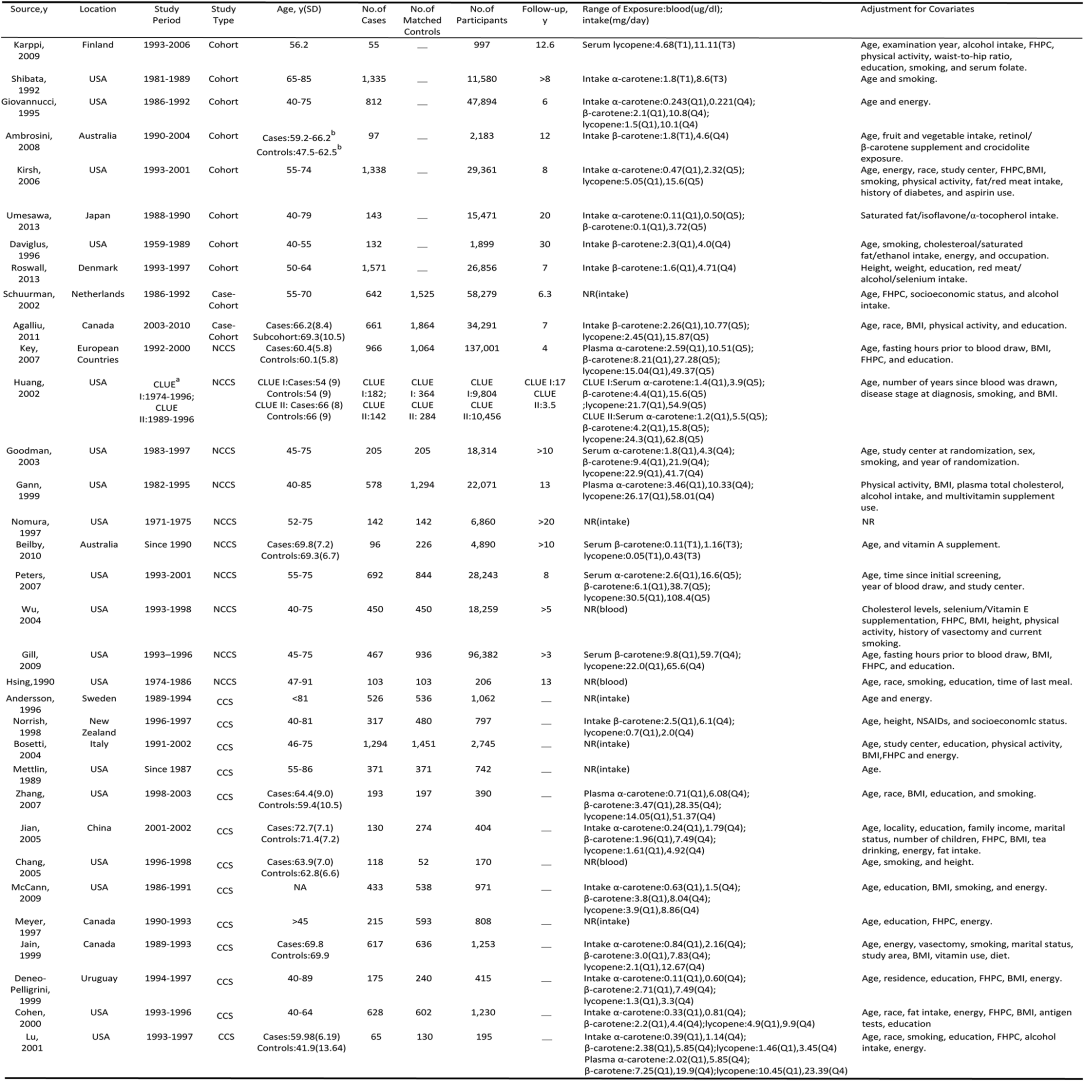
Pooled risks according to dietary carotenoids intake and its blood levels. Dietary intake of α-carotene, β-carotene, lycopene and PCa risk(left), blood levels of α-carotene, β-carotene, lycopene and PCa risk(right).

A significant inverse association was observed between dietary α-carotene intake and PCa(RR:0.81; 95% CI:0.76–0.99)(Fig 2, left). No significant statistical difference was observed between dietary β-carotene and PCa risk(RR:0.90; 95% CI:0.81–1.01). Exclusion of any individual study regarding the dietary intake of β-carotene did not change the pooled results substantially. No significant statistical difference was observed in dietary lycopene intake, with a RR of 0.88(95% CI: 0.76–1.02; I^2^ = 23.61%). However, sensitivity analysis further showed that when omitting the study conducted by Jian et al., which has a wide variation in confidence intervals and deviatesfrom the pooled trend, the results did change appreciably(RR:0.91; 95% CI:0.83–1.00) and the heterogeneity among the remaining studies decreased to 0%. Therefore, dietary lycopene intake was inversely associated with the risk of PCa.

We next explored the risk analyses stratified according to the study type, region and covariate adjustments to examine sources of study heterogeneity and the influence of potential residual confounding factors, such as age, BMI, FHPC, education, smoking, etc(Table 2).

**Table 2:** Subgroup analysis regarding the association between carotenoids consumption and PCa risk.

Subgroup*	Dietary intake(mg/day)									Blood levels(ug/dl)								
	α-carotene			β-carotene			lycopene			α-carotene			β-carotene			lycopene		
	No. of studies	Risk ratio(95%CI)	P	No. of studies	Risk ratio(95%CI)	P	No. of Studies	Risk ratio(95%CI)	P	No. of studies	Risk ratio(95%CI)	P	No. of studies	Risk ratio(95%CI)	P	No. of Studies	Risk ratio(95%CI)	P
Overall	12	0.87(0.76–0.99)	0.04	19	0.90(0.81–1.01)	0.07	13	0.88(0.76–1.02)^#^	0.083^#^	11	0.91(0.72–1.15)	0.44	13	0.96(0.81–1.14)	0.65	15	0.81(0.69–0.96)	0.015
Study type																		
Cohort(Case-cohort)	4	0.86(0.66–1.11)	0.25	8	1.02(0.92–1.14)	0.68	4	0.87(0.77–0.99)	0.029	0	-	-	0	-	-	1	0.78(0.37–1.65)	0.52
NCCS	0	-	-	0	-	-	0			8	1.01(0.86–1.19)	0.91	10	1.02(0.87–1.19)	0.81	3	0.82(0.70–0.97)	0.02
CC	8	0.86(0.74–1.01)	0.06	11	0.80(0.68–0.95)	0.01	9	0.87(0.67–1.13)	0.29	3	0.51(0.18–1.43)	0.2	3	0.62(0.40–0.93)	0.04	11	0.69(0.29–1.64)	0.4
Regions																		
Asian countries	2	0.44(0.27–0.74)	0	2	0.40(0.23–0.69)	0.001	1	0.18(0.08–0.41)	<0.01	0	-	-	0	-	-	0	-	-
North America	7	0.96(0.86–1.08)	0.49	10	0.94(0.83–1.08)	0.4	8	0.91(0.81–1.01)	0.06	10	0.88(0.67–1.12)	0.28	11	0.99(0.80–1.24)	0.94	3	0.82(0.68–1.00)	0.05
European countries	2	0.80(0.64–1.01)	0.06	5	0.93(0.80–1.08)	0.33	3	0.89(0.73–1.09)	0.27	1	1.2(0.87–1.66)	0.27	2	0.87(0.66–1.15)	0.33	12	0.75(0.49–1.17)	0.21
Australia	0	-	-	1	0.96(0.58–1.60)	0.88	0	-	-	0	-	-	0	-	-	0	-	-
Other	1	0.90(0.50–1.61)	0.72	1	1.00(0.58–1.73)	1	1	1.20(0.68–2.13)	0.53	0	-	-	0	-	-	0	-	-
Covariate adjustments																		
Adjustment for age	11	0.90(0.80–1.01)	0.08	17	0.90(0.80–1.01)	0.07	13	0.88(0.76–1.02)	0.08	8	0.95(0.71–1.29)	0.76	11	0.92 (0.78–1.09)	0.34	12	0.84(0.70–1.01)	0.07
No adjustment for age	1	0.46(0.22–0.97)	0.04	2	0.82(0.44–1.54)	0.54	0	-	-	3	0.78(0.59–1.02)	0.07	2	1.54(0.90–2.65)	0.12	3	0.73(0.50–1.06)	0.1
Adjustment for BMI	7	0.88(0.78–1.00)	0.05	7	0.81(0.65–1.01)	0.06	8	0.87(0.72–1.05)	0.16	6	0.94(0.76–1.17)	0.59	7	0.92(0.76–1.11)	0.37	8	0.77(0.66–0.91)	0
No adjustment for BMI	5	0.78(0.54–1.13)	1.19	12	0.98(0.89–1.09)	0.73	5	0.88(0.69–1.12)	0.31	5	0.79(0.46–1.35)	0.39	6	0.95(0.64–1.39)	0.77	7	0.90(0.64–1.29)	0.57
Adjustment for energy	10	0.98(0.82–1.03)	0.15	11	0.85(0.72–0.996)	0.04	10	0.89(0.74–1.08)	0.24	1	0.26(0.07–1.01)	0.05	1	0.43(0.13–1.46)	0.18	1	0.17 (0.04–0.75)	0.02
No adjustment for energy	2	0.58(0.38–0.87)	0.01	8	0.99(0.88–1.11)	0.86	3	0.82(0.66–1.02)	0.08	10	0.95(0.76–1.18)	0.61	12	0.98(0.83–1.16)	0.78	14	0.84(0.73–0.97)	0.02
Adjustment for education	7	0.81(0.68–0.96)	0.01	9	0.82(0.67–0.995)	0.04	8	0.85(0.64–1.14)	0.28	3	0.94(0.48–1.84)	0.87	6	0.84(0.69–1.03)	0.1	7	0.71(0.54–0.94)	0.02
No adjustment for education	5	0.91(0.75–1.12)	0.38	10	0.98(0.87–1.11)	0.75	5	0.89(0.89–1.00)	0.06	8	0.88(0.68–1.14)	0.33	7	1.10(0.83–1.47)	0.5	8	0.89(0.73–1.10)	0.28
Adjustment for FHPC	8	0.84(0.73–0.95)	0.01	7	0.75 (0.59–0.94)	0.01	8	0.89(0.67–1.16)	0.39	3	0.75(0.39–1.44)	0.39	5	0.87(0.70–1.07)	0.18	6	0.66 (0.48–0.93)	0.02
No adjustment for FHPC	4	0.97(0.77–1.22)	0.78	12	0.99(0.90–1.09)	0.84	5	0.84(0.74–0.97)	0.014	8	0.94(0.72–1.23)	0.67	8	1.04(0.81–1.34)	0.75	9	0.91(0.78–1.08)	0.27
Adjustment for smoking	4	0.94(0.81–1.09)	0.42	5	0.99(0.81–1.20)	0.89	4	0.95(0.82–1.10)	0.49	7	0.78(0.53–1.15)	0.21	8	0.91(0.68–1.20)	0.49	9	0.73(0.56–0.96)	0.03
No adjustment for smoking	8	0.80(0.65–0.98)	0.04	14	0.88(0.77–1.00)	0.06	9	0.85(0.69–1.05)	0.14	4	1.05(0.84–1.32)	0.67	5	1.00(0.79–1.26)	0.98	6	0.94(0.82–1.08)	0.17
Adjustment for alcohol	2	0.61(0.39–0.97)	0.04	3	1.00(0.86–1.17)	0.98	2	0.88(0.57–1.36)	0.56	2	0.55(0.21–1.47)	0.23	1	0.43(0.13–1.46)	0.18	3	0.63(0.36–1.12)	0.12
No adjustment for alcohol	10	0.90(0.79–1.02)	0.11	16	0.89(0.78–1.01)	0.06	11	0.88(0.75–1.03)	0.12	9	0.98(0.77–1.25)	0.87	12	0.98(0.83–1.16)	0.78	12	0.85(0.72–1.02)	0.07
Adjustment for PA	2	0.90(0.77–1.04)	0.15	3	0.90(0.68–1.18)	0.45	3	0.92(0.81–1.05)	0.22	2	0.74(0.55–0.98)	0.04	1	1.48(0.67–3.28)	0.33	3	0.70(0.53–0.93)	0.01
No adjustment for PA	10	0.83(0.69–1.01)	0.06	16	0.91(0.80–1.02)	0.12	10	0.86(0.69–1.07)	0.18	9	0.98(0.75–1.29)	0.89	12	0.94(0.79–1.12)	0.51	12	0.85(0.70–1.03)	0.11

Abbreviations: NCCS, nested case-control study; CCS, case-control study; BMI, body mass index; FHPC, family history of prostate cancer; PA, physical activity; NAM, No adjustment; CI, confidence interval.

*Subgroup analysis was performed in a random-effects model.

^#^Exclusion of the study conducted by Jian, et al. yielded a pooled risk ratio: 0.97(95%CI:0.83–1.00, p = 0.04) with no heterogeneity among the remaining studies(I^2^ = 0.0%,p = 0.65).

For dietary intake of α-carotene exposure, subgroup analyses indicated that the protective effect of α-carotene was more evident in Asian countries than in the North America or European countries. In addition, inverse association was more evident in studies that adjusted for education, FHPC, and alcohol compared with studies without such adjustments. β-carotene intake also exerted a protective effect on Asian populations. Overall, our stratified analyses showed that β-carotene intake has no association with PCa risk. For dietary intake of lycopene exposure, the inverse association between lycopene intake and PCa risk was evident in 4 cohort studies(RR:0.87; 95%CI:0.77–0.99).

### Blood levels of α-carotene, β-carotene, lycopene and PCa risk

Carotenoids concentrations, compared with dietary assessment, may provide a more accurate estimation of intake. However, pooled results showed that only blood levels of lycopene were significantly associated with reduced PCa risk(0.81, 0.69–0.96)(Fig 2,right). Subgroup analyses demonstrated that neither α-carotene nor β-carotene concentrations was associated with reducing the PCa risk(Table 2). The inverse association between lycopene concentrations and PCa risk was more evident in studies that adjusted for BMI, education, FHPC, smoking and physical activity compared with studies without such adjustments.

**Fig 3:**
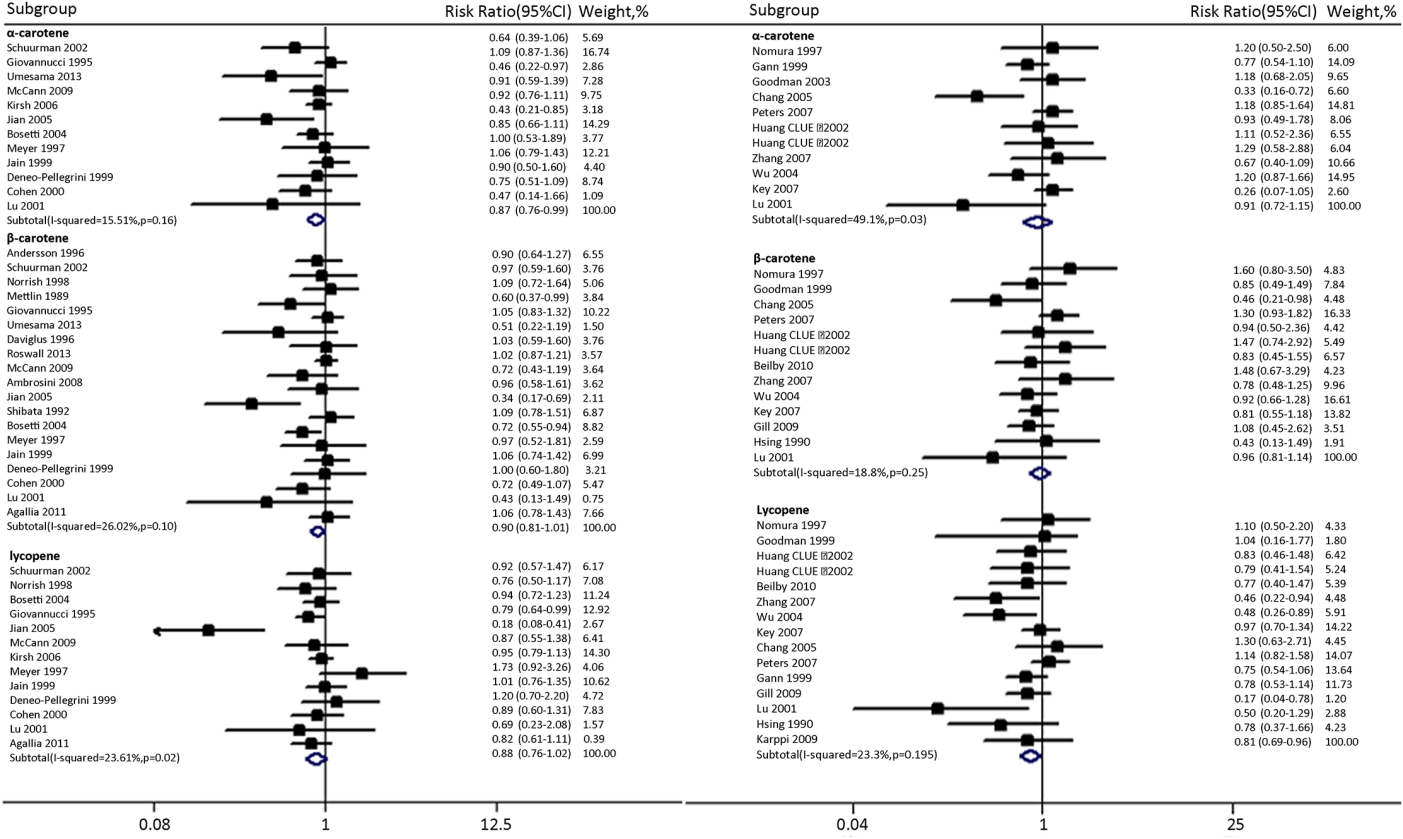
Association between blood α-carotene and lycopene levels and risk of advanced PCa. Advanced PCa was defined as stage III or IV or Gleason score ≥7.

Two studies [27, 40] and 4 studies [27, 34, 39, 40] reported the RRs of advanced PCa risk concerning blood levels of α-carotene and lycopene, respectively. However, both of them could not lower the risk of advanced PCa. RRs for blood levels of α-carotene and lycopene were 1.07(95%CI: 0.75–1.52; I^2^ = 0%) and 0.75(0.44–1.28; I^2^ = 63.2%), respectively(Fig 3).

### Dose-response analysis

**Fig 4:**
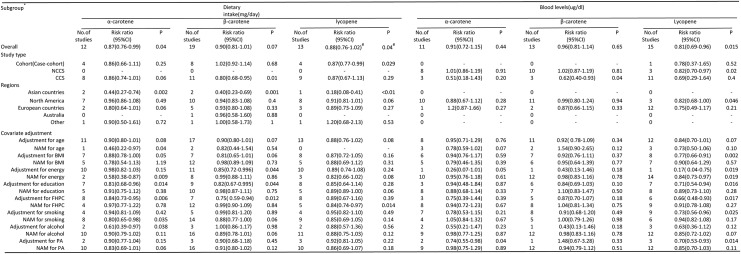
Dose-response relation plots between carotenoids consumption and risk of PCa. (A) Dietary lycopene intake(mg/day) and risk of PCa; (B) Blood lycopene levels (ug/dl) and risk of PCa; (C) Dietary α-carotene intake(mg/day) and risk of PCa. These relationships were estimated by using random-effects metaregression. Dotted lines represent the 95% CIs for the fitted trend.

With respect to the lycopene exposure, 7 studies [16, 28, 32, 41, 47, 50, 55] and 8 studies [17, 26, 27, 34, 36, 51, 52] were eligible for dose-response analysis of dietary intake and concentrations, respectively. In the cubic spline model, we showed a nonlinear association between dietary lycopene intake and risk of PCa (Fig 4A; *P*-nonlinearity = 0.014, *P*_heterogeneity_ = 0.048) and PCa risk was reduced by 3% per 1mg/day (95%CI: 0.94–0.99) increment of dietary lycopene intake. However, we showed no significant association between lycopene concentrations and risk of PCa(Fig 4B;*P*-nonlinearity = 0.24, *P*_heterogeneity_ = 0.21). With respect to the α-carotene exposure, 3 studies [16, 31, 32] were eligible for dose-response analysis, a nonlinear association between dietary α-carotene intake and risk of PCa was observed(Fig 4C; *P*-nonlinearity = 0.15, *P*_heterogeneity_ = 0.02) and PCa risk was reduced by 2% per 0.2mg/day (95% CI: 0.96–0.99) increment of dietary α-carotene intake. The dose-response relationship in terms of PCa risk was not found in either α-carotene concentrations or β-carotene(both dietary intake and concentrations)(data not shown).

## Discussion

Our meta-analysis indicated that α-carotene and lycopene, but not β-carotene, were inversely associated with the risk of PCa and both α-carotene and lycopene could not lower the risk of advanced PCa. Inverse association between α-carotene and PCa risk was augmented by adjustment for education, FHPC, and alcohol and attenuated by adjustment for age and smoking, suggesting that the association was largely mediated through education, FHPC, and alcohol, smoking and age(Table 2). Similarly, significant inverse association between lycopene and PCa risk was largely mediated through FHPC. With respect to carotenoids concentrations, inverse association between lycopene and PCa risk was largely mediated through education, FHPC, smoking and physical activity.

Lycopene is found to be a more efficient antioxidant than β-carotene, α-carotene, and α-tocopherol [56]. Among all major carotenoids, lycopene and tomato products have been most extensively studied [10]. A meta-analysis [57] including 11 case-control studies and 10 cohort studies showed a modest, significant inverse relation between dietary intake of lycopene and PCa risk in cohort studies. However, the CUP report concludes that the link between PCa risk and foods containing lycopene has been downgraded from strong evidence of a decreased risk, to no conclusion possible [20]. This updating is based on a considerable amount of global research focusing on specific types of PCa, for example, fatal, advanced and early (non-advanced) prostate cancers rather than grouping all prostate cancers together. However, this does not mean that no link exists, variations in diagnosis and classifications of the PCa has made the link more difficult to see. In addition, the CUP report has not adjusted the confounding variables when interpreting the evidence. Even more important, frequent lycopene intake may reduce PCa risk via multiple mechanisms. Rafi, et al. informed that lycopene attenuates PCa by modulating the expression of growth and survival associated genes, e.g. CDK7, BCL2, EGFR, and IGF-1R [58]; in addition, lycopene can inhibit PCa cell proliferation via PPARγ-LXRα-ABCA1 pathway [59]; Further, Zu, et al. [60] found that dietary intake of lycopene is associated with reduced risk of lethal PCa and with a lesser degree of angiogenesis in the tumor.

In 1995, van Poppel and Goldbohm [61] summarized all previous studies exploring the risk between β-carotene intake and all types of tumors. They concluded that the association appears most consistent for lung and stomach cancer, whereas the association seems inconsistent for breast and PCa. More recently, a meta-analysis [55] of randomized controlled trials demonstrated that no effect of β-carotene supplementation was observed in the incidence of PCa(RR, 0.99; 95% CI, 0.91–1.07). Although there was *in vitro* study indicating that β-carotene may act as a growth-inhibitory agent in PCa cells by modulating the caveolin-1 pathway [62], subsequent big data from the Alpha-Tocopherol, Beta-Carotene Cancer Prevention(ATBC) Study, a randomized, double-blind, placebo-controlled trial, suggested that serum β-carotene, serum retinol, and supplemental β-carotene had no apparent effects on PCa patients’ survival [63]. Our pooled and stratified analyses suggested that neither dietary β-carotene intake nor its concentrations was related to the PCa risk, moreover, the null dose-response relationship(data not shown) supported this conclusion. Also, the CUP report concludes that there is strong evidence that consuming β-carotene (either through food or supplements) is unlikely to have a substantial effect on the risk of PCa [20]. The studies on which the CUP report based are all cohort studies which should be given high priority.

Although α-carotene is chemically similar to β-carotene, α-carotene has higher biological activity in inhibiting the proliferation of human neuroblastoma cells [64] and liver carcinogenesis [65]. Besides, serum α-carotene concentrations are inversely associated with risk of death from all causes, cardiovascular disease, cancer, and all other causes [66]. Our pooled analysis indicated a significant inverse relation between α-carotene intake and PCa risk, which was further verified by dose-response analysis(Fig 4C).

It was reported that in Caucasians, the frequencies of fusion of *TMPRSS2*:*ERG*, the most common known genetic alteration in PCa, are 50%–70% [67], while in Asian patients the frequencies are lower than 20% [68]. Mao et al. revealed that low-level expression of *PTEN* is detected in 69.8% (111/159) of UK PCa samples, but only in 34% (31/91) of Chinese samples [69]. Whereas, *RAS*-*RAF*-*MAPK* pathway mutants are much more frequently found in Asian PCa patients than patients from Western countries [70]. All these abnormal genes are likely to contribute to susceptibility to PCa in different ethnic groups. Broccoli, green beans, green peas, spinach, turnip greens, leaf lettuce, the main source of vegetables for China adults, are rich in α-carotene [66]. Specially, our study showed that α-carotene exerted a greater protective effect on Asians. All of these suggested α-carotene may attenuate the PCa susceptibility by interacting with the genetic or environmental factors. However, the fundamental researches that aim at elucidating the links between α-carotene intake and PCa risk are lacking. Altogether, this may shed fresh new light on α-carotene’s mode of action.

Compared with a meta-analysis [57] conducted in 2004, we put emphasis on the single-ingredient of carotenoids rather than raw tomato or its processed products, which may provide more detailed and accurate assessment on the links between carotenoids consumption and PCa risk. Furthermore, dose-response analysis was utilized for the first time to reveal the links between carotenoids consumption and PCa risk. However, our study was subject to one inconsistency: significant association was only found in dietary intake, but not its concentrations. There are 3 case-control studies involving in exploring the links between α-carotene concentrations and PCa risk(Table 2). These retrospective studies may have biased the pooled results.

Not all included studies have adjusted for some important covariates, which are major concerns in our study, may have confounded these associations. For example, the inverse association between dietary α-carotene intake and PCa risk was more evident in studies without adjustment for smoking compared with studies with such adjustment(Table 2), suggesting more high-quality observational studies are warranted to verify the effect of dietary α-carotene intake on PCa risk. In addition, although subgroup analyses were performed, heterogeneity could not wholly explained by the remaining variables, suggesting that other unknown factors are introduced. Furthermore, in terms of α-carotene and lycopene consumption, not all studies were eligible for dose-response analysis, indicating that risks corresponding to dose increments are partially right.

In summary, findings from our study indicate that α-carotene and lycopene, but not β-carotene, are inversely associated with the risk of PCa. However, both α-carotene and lycopene can not lower the risk of advanced PCa. Our results, if replicated in other cohort studies and populations, suggest a need for clinical research into the health benefits of α-carotene and lycopene supplementation.

## Supporting Information

S1 PRISMA Checklist PRISMA checklist. (DOC)

S1 Table Methodological quality assessment based on the NOS. (DOCX)
