# Correction: Effect of Carotene and Lycopene on the Risk of Prostate Cancer: A Systematic Review and Dose-Response Meta-Analysis of Observational Studies

**DOI:** 10.1371/journal.pone.0140415

**Published:** 2015-10-08

**Authors:** Yulan Wang, Ran Cui, Yuanyuan Xiao, Juemin Fang, Qing Xu

Fig 2 is incorrect. The image that appears as Fig 2 is a duplicate of Table 1. Please view the correct Fig 2 below.

**Fig 2:**
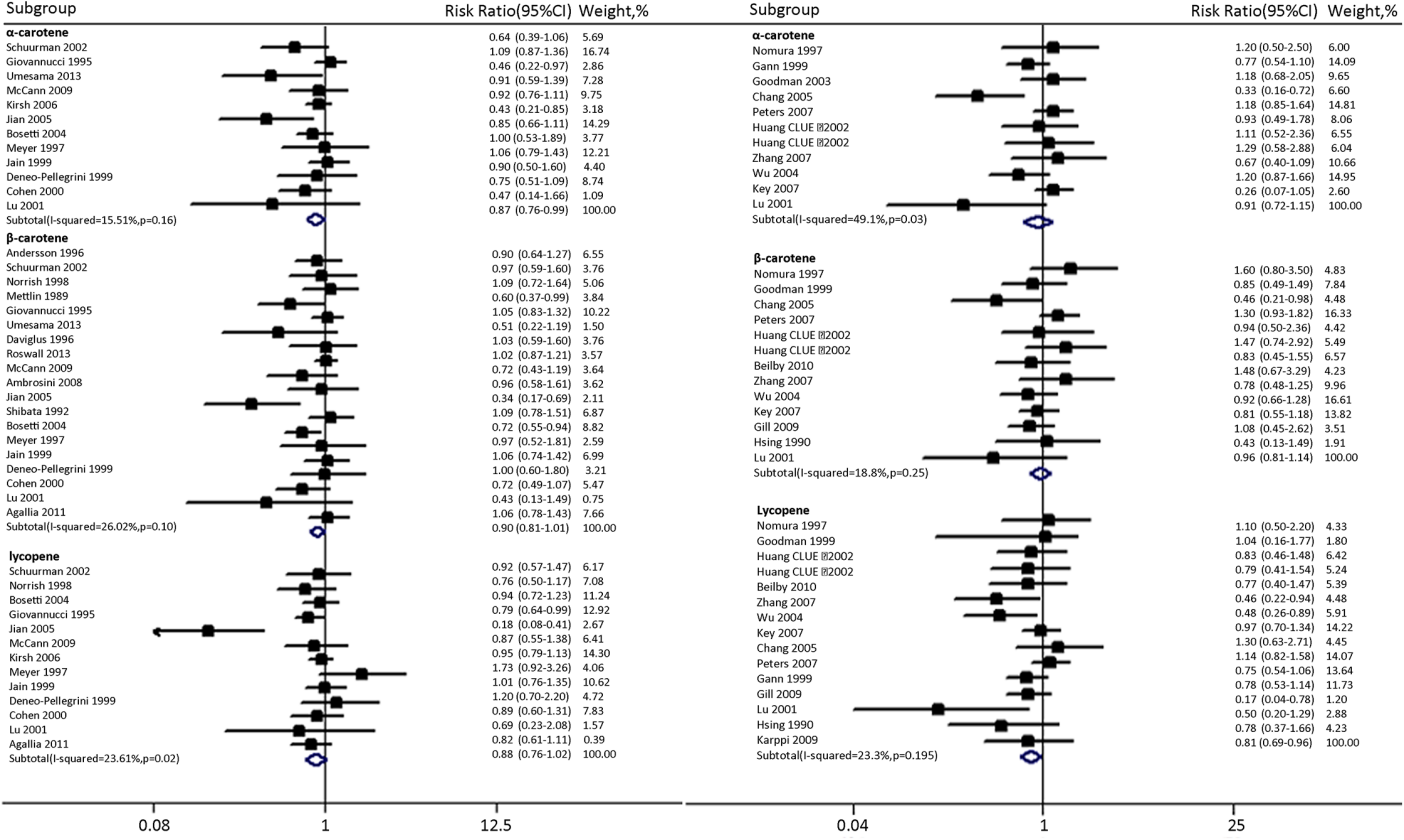
Pooled risks according to dietary carotenoids intake and its blood levels. Dietary intake of α-carotene, β-carotene, lycopene and PCa risk(left), blood levels of α-carotene, β-carotene, lycopene and PCa risk(right).

Fig 3 is incorrect. The image that appears as Fig 3 is a duplicate of the correct Fig 2. Please view the correct Fig 3 below.

**Fig 3:**
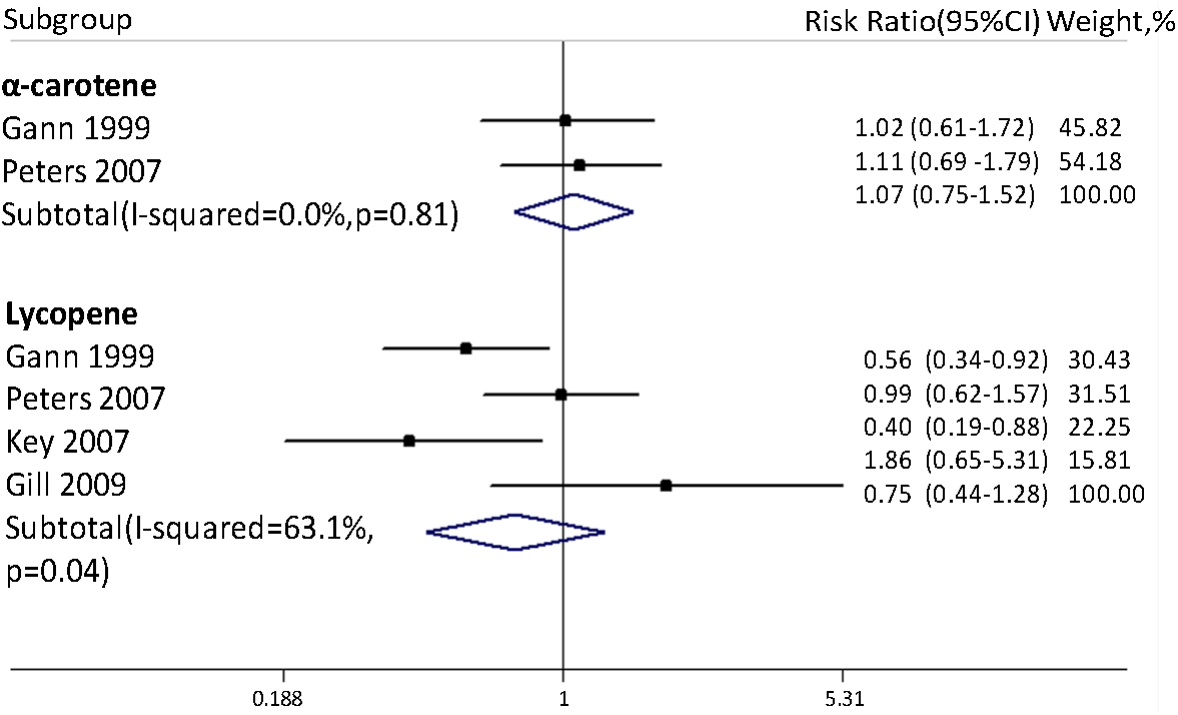
Association between blood α-carotene and lycopene levels and risk of advanced PCa. Advanced PCa was defined as stage III or IV or Gleason score ≥7.

Fig 4 is incorrect. The image that appears as Fig 4 is a duplicate of Table 2. Please view the correct Fig 4 below.

**Fig 4:**
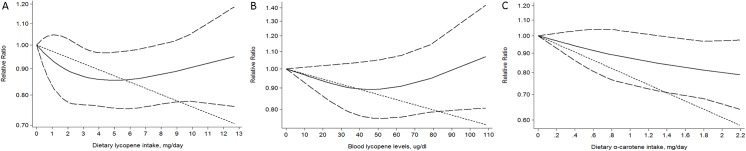
Dose-response relation plots between carotenoids consumption and risk of PCa. (A) Dietary lycopene intake(mg/day) and risk of PCa; (B) Blood lycopene levels (ug/dl) and risk of PCa; (C) Dietary α-carotene intake(mg/day) and risk of PCa. These relationships were estimated by using random-effects metaregression. Dotted lines represent the 95% CIs for the fitted trend.
