# Supplementary material for: Effect of Carotene and Lycopene on the Risk of Prostate Cancer: A Systematic Review and Dose-Response Meta-Analysis of Observational Studies
Source: PLoS One. 2015 Sep 15;10(9):e0137427. doi: 10.1371/journal.pone.0137427 (PMC4570783; doi:10.1371/journal.pone.0137427)
Supplement: S1 Table — (DOCX) [file pone.0137427.s002.docx]

| Source | Selection | | | | Comparability^5^ | Exposure | | | Total^9^ |
| --- | --- | --- | --- | --- | --- | --- | --- | --- | --- |
|  | Definition^1^ | Representativeness^2^ | Selection^3^ | Definition^4^ |  | Ascertainment^6^ | Method^7^ | Rate^8^ |  |
| Key,2007 | ☆ | ☆ | ☆ | ☆ | ☆☆ | ☆ | ☆ | 0 | 8 |
| Huang,CLUE I 2002 | ☆ | ☆ | ☆ | ☆ | ☆☆ | ☆ | ☆ | 0 | 8 |
| Huang,CLUE II 2002 | ☆ | ☆ | ☆ | ☆ | ☆☆ | ☆ | ☆ | 0 | 8 |
| Goodman,2003 | ☆ | ☆ | ☆ | ☆ | 0 | ☆ | ☆ | 0 | 6 |
| Gann,1999 | ☆ | 0 | ☆ | ☆ | ☆ | ☆ | ☆ | 0 | 6 |
| Nomura,1997 | ☆ | 0 | ☆ | 0 | 0 | ☆ | ☆ | 0 | 4 |
| Shibata,1992 | ☆ | ☆ | ☆ | 0 | ☆ | ☆ | ☆ | ☆ | 7 |
| Andersson,1996 | ☆ | ☆ | ☆ | ☆ | ☆ | ☆ | ☆ | 0 | 7 |
| Schuurman,2002 | ☆ | ☆ | ☆ | ☆ | ☆☆ | ☆ | ☆ | ☆ | 9 |
| Norrish,1998 | ☆ | ☆ | ☆ | ☆ | ☆ | ☆ | ☆ | 0 | 7 |
| Bosetti,2004 | ☆ | ☆ | 0 | ☆ | ☆☆ | ☆ | ☆ | ☆ | 8 |
| Mettlin,1989 | ☆ | ☆ | ☆ | ☆ | ☆ | ☆ | ☆ | 0 | 7 |
| Giovannucci,1995 | ☆ | ☆ | ☆ | ☆ | ☆☆ | ☆ | ☆ | 0 | 8 |
| Ambrosini,2008 | ☆ | ☆ | ☆ | ☆ | ☆☆ | ☆ | ☆ | 0 | 8 |
| Beilby,2010 | ☆ | ☆ | ☆ | ☆ | ☆☆ | ☆ | ☆ | 0 | 8 |
| Zhang,2007 | ☆ | 0 | 0 | ☆ | ☆☆ | ☆ | ☆ | 0 | 6 |
| Wu,2004 | ☆ | ☆ | ☆ | ☆ | ☆☆ | ☆ | ☆ | ☆ | 9 |
| Kirsh,2006 | ☆ | ☆ | ☆ | ☆ | ☆☆ | ☆ | ☆ | 0 | 8 |
| Jian,2005 | ☆ | 0 | 0 | ☆ | ☆☆ | ☆ | ☆ | ☆ | 7 |
| Umesawa,2013 | 0 | ☆ | ☆ | ☆ | ☆☆ | ☆ | ☆ | 0 | 7 |
| Daviglus,1996 | ☆ | ☆ | ☆ | ☆ | ☆☆ | ☆ | ☆ | 0 | 8 |
| Roswall,2013 | ☆ | ☆ | ☆ | ☆ | ☆☆ | ☆ | ☆ | 0 | 8 |
| Chang,2005 | ☆ | ☆ | 0 | ☆ | ☆ | ☆ | ☆ | 0 | 6 |
| McCann,2009 | ☆ | ☆ | ☆ | ☆ | ☆☆ | ☆ | ☆ | 0 | 8 |
| Peters,2007 | ☆ | ☆ | ☆ | ☆ | ☆☆ | ☆ | ☆ | 0 | 8 |
| Gill,2009  Deneo-Pelligrini**,**1999  Cohen,2000  Lu,2001  Meyer,1997  Jain,1999  Karppi,2009  Agalliu,2011  Hsing,1990 | ☆  ☆  ☆  ☆  ☆  ☆  ☆  ☆  ☆ | ☆  ☆  ☆  ☆  ☆  ☆  ☆  ☆  ☆ | ☆  0  ☆  0  ☆  ☆  ☆  ☆  ☆ | ☆  ☆  ☆  ☆  ☆  ☆  ☆  ☆  ☆ | ☆☆  ☆☆  ☆☆  ☆☆  ☆☆  ☆☆  ☆☆  ☆☆  ☆☆ | ☆  ☆  ☆  ☆  ☆  ☆  ☆  ☆  ☆ | ☆  ☆  ☆  ☆  ☆  ☆  ☆  ☆  ☆ | 0  0  ☆  0  ☆  ☆  0  0  0 | 8  7  9  7  9  9  8  8  8 |

|  |
| --- |
|  |
| ^1^Adequate definition of cases(0,1star)  ^2^Consecutive or obviously representative series of cases (0,1)  ^3^Selection of controls: Community controls (0,1)  ^4^Definition of controls: No history of disease (endpoint) (0,1)  ^5^Study controls for the most important factor or any additional factor(0,1,2)  ^6^Secure record (eg surgical records) (0,1)  ^7^Same method of ascertainment for cases and controls(0,1)  ^8^Same non-response rate for both groups(0,1)  ^9^Total: minimum equals 1; maximum equals 9 stars |
|  |
|  |
|  |
|  |
|  |
|  |
|  |
